# Drug Prescription in Older Swiss Men and Women Followed in Family Medicine

**DOI:** 10.1007/s40801-019-00175-6

**Published:** 2019-12-17

**Authors:** David Schnegg, Nicolas Senn, Olivier Bugnon, Joëlle Schwarz, Yolanda Mueller

**Affiliations:** 1 University of Lausanne, Lausanne, Switzerland; 2 Department of Family Medicine/Center for Primary Care and Public Health (Unisanté), University of Lausanne, Lausanne, Switzerland

## Abstract

**Background:**

We sought to estimate the prevalence of polypharmacy, the most prevalent drug classes involved, and the prevalence and type of potentially inappropriate prescribing among older male and female patients in family medicine.

**Methods:**

We conducted a secondary analysis of baseline data from a pragmatic cluster-randomised trial on the efficacy of a screening and management tool for geriatric syndromes among older community-dwelling patients (aged ≥ 75 years) included by 42 family physicians. Information on drug prescription and clinical diagnoses (International Classification of Primary Care—2nd Edition [ICPC-2] coded) were extracted manually from medical records. The prevalence of polypharmacy, defined as the use of at least five permanent oral or parenteral drugs, and of potentially inappropriate medications (PIMs), identified according to 2015 updated Beers criteria, were compared between men and women.

**Results:**

We included 429 patients (269 women and 160 men; mean age 82.9 and 81.8 years, respectively). Polypharmacy was found in 59.9% of them. Analgesics, antithrombotic agents and agents acting on the renin-angiotensin system were the most frequently prescribed drug categories. Three-quarters of patients (76.7%) were prescribed at least one PIM according to Beers criteria, without difference by sex/gender (*p *= 0.760). The most frequent PIMs were proton-pump inhibitors used for > 8 weeks, diuretics, benzodiazepines, aspirin for primary prevention, and chronic use of non-steroidal anti-inflammatory drugs. Prescription patterns markedly differed by sex/gender, but the number and patterns of inappropriate prescriptions were comparable overall.

**Interpretation:**

Both polypharmacy and PIMs were very common in older patients followed regularly in family medicine in Switzerland. Interestingly, most PIMs involved only a limited number of medication classes.

**Trial registration:**

Clinicaltrials.gov NCT 02618291.

**Electronic supplementary material:**

The online version of this article (10.1007/s40801-019-00175-6) contains supplementary material, which is available to authorized users.

## Key Points

**Table:** 

Older patients, both male and female, followed in family medicine are prescribed a high number of drugs, but different drug classes are prescribed to older men than to older women.
Potentially inappropriate prescribing is very common in both older men and older women.
Most potentially inappropriate medications are concentrated among a few drug classes.
Targeted deprescription advice, differentiated by sex/gender and focusing on the most prevalent drug classes, could simplify deprescribing for family physicians.

## Introduction

Polypharmacy and inappropriate prescribing are important clinical challenges, especially among older patients, who often have multiple chronic conditions. Polypharmacy is heterogeneously defined in studies as the use of multiple medications by the patient, although most studies agree on a threshold of at least five medications [1]. In Switzerland, one-quarter of community-dwelling patients aged > 65 years self-report taking five or more drugs [2], although higher rates have been reported based on claims data [3, 4].

Polypharmacy is associated with potentially inappropriate medications (PIMs) [5]. The prevalence of PIM varies according to age, context (community dwelling vs. care home) and the criteria used to define it [6–8]. Swiss data in community-dwelling patients aged > 65 years reported a prevalence of PIM around 20% [3, 9, 10], increasing to 74% in nursing home residents [4]. However, these studies were limited to claims data or conducted in specific populations, and clinical information to estimate inappropriate prescribing was often weak. The most prevalent classes of PIM were psycholeptic agents, followed by sex hormones and genital system modulators, psychoanaleptics, anti-inflammatory and anti-rheumatic products and cardiac therapy [3]. Still, detailed data on prescriptions patterns, polypharmacy and PIM remain limited in Switzerland, especially for patients followed-up in family medicine.

Sex/gender differences have been reported in the prevalence of both polypharmacy and PIM. In the adult population, men are prescribed fewer drugs than are women, even after excluding sex/gender-related morbidity, although the difference decreases with age [11]. Among older populations, evidence on gender difference in polypharmacy varies, but older women seem to receive more PIMs [8, 12–14]. When studies do report gender differences, they most often lack further exploration or explanation of the gender discrepancy. Hofer-Dückelmann [15] explored the reasons for higher rates of polypharmacy in older women, highlighting the gendered differences in attitudes towards drug intake: the female propensity to see a physician and discuss problems, family responsibility and caregiving activities, provider–patient relationship, education, social deprivation and self-rated health. Differences in drug prescriptions have been studied in the field of cardiovascular prevention, with a Swedish study observing that older women were more likely to be treated with diuretics and nitro-glycerine, whereas older men with diabetes were more likely to receive antihyperglycaemic drugs [16]. Other studies have shown that older women receive more psychotropic medications [17–19]. Until now, sex/gender differences in drug prescriptions have not been explored in the Swiss context.

This study aims to provide insight into drug prescription patterns in male and female older patients followed in family medicine in the French-speaking part of Switzerland. The objectives were to estimate the prevalence of polypharmacy, the most prevalent drug classes, the prevalence of PIM and type and, last, the association between polypharmacy/PIMs and the sex/gender of patients.

## Method

### Study Design

We conducted a secondary analysis of baseline data from a pragmatic cluster-randomised trial on the efficacy of a screening and management tool for geriatric syndromes in family medicine (NCT 02618291). In this trial, 42 private family medicine practices in western Switzerland, selected according to their willingness to participate in the trial, included at least ten community-dwelling patients (aged ≥ 75 years), randomly selected among routinely followed patients (at least two visits in the past year) between September 2016 and January 2018.

### Drug Use and Clinical Information

Information on drug prescription and clinical diagnosis was extracted manually from paper or electronic medical records by a trained research assistant and entered into a standardised and pretested case report form. Designation was matched with corresponding anatomical therapeutic chemical (ATC) code classification [20] using a predefined list of 2628 drugs that were commercially available in Switzerland. Drugs were categorised in the corresponding second-level ATC class. Only oral or parenteral drugs were considered for this analysis. We distinguished drugs taken continuously from occasional medication based on the information recorded as comments in the case report form. For example, mentions of “stand-by treatment”, conditional use (“in case of”) and limited time (“until”) were considered occasional treatment. Continuous use was considered the default prescription in the absence of comments. Patients’ chronic conditions present in the medical file were coded by the same study staff according to a predefined list of 75 diagnoses based on International Classification of Primary Care—2nd Edition (ICPC-2; Wonca International Classification Committee) [21]. Polypharmacy was defined as the prescription of at least five permanent oral or parenteral drugs [22].

### Potentially Inappropriate Medications (PIMs)

PIMs were identified and divided into five sections according to 2015 updated Beers criteria [6] using the ICPC-2 diagnosis and ATC classification: medication to avoid in most older patients; drug–disease or drug–syndrome interactions; drugs to be used with caution in older patients; drug–drug interactions meaningful in a geriatric setting; and, finally, drugs that should be avoided or the dose reduced with impaired renal function. The last two sections were added with the 2015 update, so only the first three sections were used to assess the total number of PIMs to enable comparison with previous studies.

### Sex/Gender

Patient sex/gender categorisation was based on the information available in the medical record as recorded by the physician. Because we could not disentangle the effects of sex (understood as biological characteristics) and gender (socially constructed), we refer to the combined effect of sex/gender [23, 24].

### Data Analysis

A Wilcoxon rank-sum test was used to assess whether a difference existed in the number of medications between men and women. Proportions by sex/gender were compared with Chi squared and Fisher’s exact tests. We used logistic regression to estimate the odds ratios (ORs) of the prescription of different drug classes by sex/gender. Considering that patients were recruited via their physicians, we used a mixed logistic regression model with a random intercept by physician to adjust the ORs and compared the added value of adding the random intercept by likelihood ratio tests. *p* values < 0.05 were considered significant. Stata software (version 14, College Station, TX, USA) was used to analyse the data.

## Results

In total, 42 general practitioners (18 women, 24 men) participated in the trial, each enrolling a median number of 11 patients (interquartile range [IQR] 7–12). Final data consisted of 429 patients with a median age of 82 years (IQR 78–86), 62.7% of whom were female. Table 1 lists the sociodemographic and clinical characteristics of the included patients. Women were slightly older (82.9 vs. 81.8 years; *p *= 0.021) and were more frequently living on their own (64.8 vs. 26.4%; *p *< 0.001), receiving home-based care (21.3 vs. 11.3%; *p *= 0.009), had a lower education level (*p *< 0.001) and were less likely to drive (35.9 vs. 76.9%; *p *< 0.001). The number of ICPC-2 diagnoses was comparable between men and women (*p *= 0.194). The proportion of men, compared with women, with at least one condition reported by ICPC-2 chapter was comparable for most chapters, with the exception of eye conditions (14.1 vs. 25.6%; *p *= 0.003), musculoskeletal conditions (66.9 vs. 42.5%; *p *< 0.001) and conditions of the genital system (14.1 vs. 34.4%; *p *< 0.001).

**Table 1:** Sociodemographic and clinical characteristics

Characteristic	Women (*N *= 269)	Men (*N *= 160)	*P* value
Age (years)	82.9 ± 5.2	81.8 ± 4.5	0.021
Living alone	169 (64.5) (***N ***= 262)	42 (26.3) (***N ***= 160)	< 0.001
Driving a car	95 (35.9) (***N ***= 265)	123 (76.9) (***N ***= 160)	< 0.001
Receiving home-based care	57 (21.2) (***N ***= 269)	18 (11.3) (***N ***= 160)	0.009
Receiving help from other caregivers	64 (25.3) (***N ***= 253)	24 (16.0) (***N ***= 150)	0.029

	*N *= 249	*N *= 149	
Education (degree reached)			< 0.001
Did not finish primary school	7 (2.8)	0 (0.0)	
Primary school	85 (34.1)	28 (18.8)	
Secondary school	42 (16.9)	11 (7.4)	
Professional degree	86 (34.5)	57 (38.3)	
Higher education (university or equivalent)	29 (11.7)	53 (35.6)	

	*N *= 269	*N *= 160	
Median (interquartile range) number of chronic conditions	4 (2–5)	4 (3–6)	0.194
Chronic conditions by ICPC-2*chapter			
General	17 (6.3)	8 (5.0)	0.573
Blood	34 (12.6)	22 (13.8)	0.741
Digestive system	77 (28.6)	47 (29.4)	0.868
Eye	38 (14.1)	41 (25.6)	0.003
Ear	34 (12.6)	23 (14.4)	0.609
Cardiovascular	232 (86.3)	145 (90.6)	0.179
Musculoskeletal	180 (66.9)	68 (42.5)	< 0.001
Neurological	74 (27.5)	36 (22.5)	0.251
Psychological	87 (32.3)	53 (33.1)	0.867
Respiratory	38 (14.1)	33 (20.6)	0.080
Skin	44 (16.4)	26 (16.3)	0.977
Endocrine/metabolic and nutritional	125 (46.5)	66 (41.3)	0.293
Urological	75 (27.9)	43 (26.9)	0.821
Genital	38 (14.1)	55 (34.4)	< 0.001

Data are presented as mean ± standard deviation or *N* (%) unless otherwise indicated

*ICPC International Classification of Primary Care—2nd Edition; Wonca International Classification Committee

### Polypharmacy and Drug Classes by Sex/Gender

Patients were prescribed a median of seven drugs (IQR 5–10), or five drugs (IQR 3–8) when occasional medication was excluded, with no differences between men and women (*p *= 0.469 and *p *= 0.636, respectively; data not shown). The prevalence of polypharmacy (defined as at least five permanent drugs) was 59.9% (61.9% in men; 58.7% in women; *p *= 0.521). Table 2 lists the most frequent drug classes used. Analgesics and antithrombotic agents were prescribed to more than one-half of patients. Agents acting on the renin-angiotensin system (48.7%), mineral supplements (44.3%) and lipid-modifying agents (39.9%) were the next most frequent drug classes, followed by psycholeptics (26.6%) and drugs for acid-related disorders (26.3%). Women were more likely to be prescribed mineral supplements (54.3 vs. 27.5%; OR 3.12 [95% confidence interval {CI} 2.05–4.77]), psychoanaleptics (28.3 vs. 16.9%; OR 1.94 [95% CI 1.19–3.17]) and thyroid therapy (16.0 vs. 5.6%; OR 3.37 [95% CI 1.60–7.10]), whereas men received more prescriptions for antithrombotic drugs (62.5 vs. 43.1%; OR 0.45 [95% CI 0.30–0.68]), lipid-modifying agents (49.4 vs. 34.2%; OR 0.53 [95% CI 0.36–0.79]), urologicals (24.4 vs. 5.6%; OR 0.18 [95% CI 0.10–0.35]) and drugs used in diabetes (19.4 vs. 10.0%; OR 0.46 [95% CI 0.27–0.81]). Four drug classes were frequently prescribed for intermittent use: analgesics, psycholeptics, anti-inflammatory and antirheumatic products and drugs for constipation. A significant variation of the prescription by physician, estimated by adding a random intercept in the model, was found for agents acting on the renin-angiotensin system (*p *= 0.007), lipid-modifying agents (*p *< 0.001), β-blocking agents (*p *= 0.027) and vitamins (*p *< 0.001).

**Table 2:** Oral and parenteral drug classes prescribed to patients aged ≥ 75 years followed in primary care and included in the study, by sex/gender

Drug class (ATC)	Class name	All drugs (intermittent use included)	Continuous use only					
		Total users (*N *= 429)	Total users (*N *= 429)	Women (*N *= 269)	Men (*N *= 160)	OR^a^ (95% CI)	AdjOR (95% CI)	*p* value for cluster effect
N02	Analgesics	236 (55.0)	82 (19.1)	54 (20.1)	26 (16.3)	1.29 (0.77–2.17)	1.36 (0.79–2.35)	**0.017**
B01	Antithrombotic agents	224 (52.2)	217 (50.6)	116 (43.1)	100 (62.5)	**0.45** (**0.30–0.68**)	0.45 (0.30–0.68)	0.436
C09	Agents acting on the RAS	212 (49.4)	209 (48.7)	125 (46.5)	80 (50.0)	0.87 (0.59–1.28)	0.84 (0.56–1.28)	**0.007**
A12	Mineral supplements	194 (45.2)	190 (44.3)	146 (54.3)	44 (27.5)	**3.13** (**2.05–4.77**)	3.27 (2.10–5.08)	0.074
C10	Lipid-modifying agents	173 (40.3)	171 (39.9)	92 (34.2)	79 (49.4)	**0.53** (**0.36–0.79**)	**0.49** (**0.32–0.76**)	**< 0.001**
N05	Psycholeptics	167 (38.9)	114 (26.6)	75 (27.9)	38 (23.8)	1.24 (0.79–1.95)	1.23 (0.78–1.95)	0.372
A02	Drugs for acid-related disorders	145 (33.8)	113 (26.3)	43 (26.9)	69 (25.7)	0.94 (0.60–1.46)	0.95 (0.60–1.51)	0.126
C07	β-blocking agents	134 (31.2)	133 (31.0)	80 (29.7)	53 (33.1)	0.85 (0.56–1.30)	0.81 (0.52–1.26)	**0.027**
M01	Anti-inflammatory and antirheumatic products	113 (26.3)	60 (14.0)	38 (14.1)	22 (13.8)	1.03 (0.59–1.82)	1.05 (0.59–1.88)	0.196
N06	Psychoanaleptics	109 (25.4)	103 (24.0)	76 (28.3)	27 (16.9)	**1.94** (**1.19–3.17**)	1.94 (1.18–3.17)	0.459
C03	Diuretics	101 (23.5)	98 (22.8)	60 (22.3)	38 (23.8)	0.92 (0.58–1.47)	0.94 (0.58–1.51)	0.095
A06	Drugs for constipation	104 (24.2)	57 (13.3)	35 (13.0)	22 (13.8)	0.94 (0.53–1.66)	0.94 (0.52–1.70)	0.113
A11	Vitamins	72 (16.8)	71 (16.6)	47 (17.5)	24 (15.0)	1.20 (0.70–2.05)	1.33 (0.73–2.40)	**< 0.001**
C08	CCBs	71 (16.6)	65 (15.2)	45 (16.7)	20 (12.5)	1.41 (0.80–2.48)	NA	
G04	Urologicals	58 (13.5)	53 (12.4)	15 (5.6)	39 (24.4)	**0.18** (**0.10–0.35**)	NA	
A10	Drugs used in diabetes	58 (13.5)	58 (13.5)	27 (10.0)	31 (19.4)	**0.46** (**0.27–0.81**)	NA	
H03	Thyroid therapy	53 (12.4)	52 (12.1)	43 (16.0)	9 (5.6)	**3.37** (**1.60–7.10**)	3.51 (1.63–7.57)	0.115
C01	Cardiac therapy	51 (11.9)	34 (7.9)	20 (7.4)	14 (8.8)	0.84 (0.41–1.71)	0.79 (0.37–1.66)	0.093
B03	Anti-anaemic preparations	41 (9.6)	40 (9.3)	16 (10.0)	25 (9.3)	0.92 (0.48–1.78)	1.06 (0.50–2.23)	**< 0.001**
C05	Vasoprotectives	36 (8.4)	36 (8.4)	26 (9.7)	7 (4.4)	2.34 (0.99–5.52)	2.52 (1.03–6.16)	0.157
M04	Antigout preparations	34 (7.9)	31 (7.2)	13 (4.8)	17 (10.6)	0.43 (0.20–0.90)	0.42 (0.19–0.90)	0.213

Drugs classed according to ATC code, second-level class. Odds ratios of class prescription by sex/gender from a logistic regression model, raw and after adding a random intercept by physician. Restricted to drug classes prescribed to ≥ 10% of either male or female patients

Bold formatting indicates statistical significance

*AdjOr* adjusted OR, *ATC* anatomical therapeutic chemical, *CCBs* calcium channel blockers, *CI* confidence interval, *NA* not applicable: non-convergence of mixed logistic regression model, *OR* odds ratio, *RAS* renin-angiotensin system

^a^Baseline: Men

### PIM and Sex/Gender

The percentage of patients with at least one PIM was 76.7%, with a median number of two PIMs per patient (IQR 1–3). Table 3 lists the ten most prevalent PIMs, representing 93.8% of all identified PIMs, along with the rationale for the recommendation. The most frequent medications to avoid for most older adults were proton-pump inhibitors (PPIs) prescribed for a duration > 8 weeks (23.1% of patients), benzodiazepines (21.5%), chronic use of oral non-cyclooxygenase-selective non-steroidal anti-inflammatory drugs (NSAIDs) (16.8%) and nonbenzodiazepine/benzodiazepine receptor agonist hypnotics (9.8%). The most frequent drugs that should be used with caution in most older adults included diuretics (28.4% of patients), aspirin for primary prevention of cardiac events (19.8%), vasodilators (15.8%) and selective serotonin reuptake inhibitors (SSRIs) (12.4%).

**Table 3:** The ten most prevalent PIMs according to the 2015 updated Beers criteria, and summary of the rationale for the recommendation

Beers criteria item	ATC class	*N* (%)	Rationale
Diuretics	C03	122 (28.4)	Use with caution; may exacerbate or cause SIADH or hyponatraemia
Proton-pump inhibitors	A02BC	99 (23.1)	Avoid scheduled use for > 8 weeks unless in high-risk patients
Benzodiazepines	N05BA12, N05CD04, N05BA06, N05BA56, N05BA04, N05CD07, N05CD05, N05BA05, N05BA02, N03AE01, N05BA01, N05BA17, N05CD01, N05CD10	92 (21.5)	Avoid; older adults have increased sensitivity to benzodiazepines and decreased metabolism of long-acting agents. In general, all benzodiazepines increase the risk of cognitive impairment, delirium, falls, fractures and motor vehicle crashes in older adults
Aspirin for primary prevention of cardiac events	B01AC	85 (19.8)	Use with caution in patients aged ≥ 80 years
Non-cyclooxygenase-selective NSAIDs, oral	M01A	71 (16.6)	Avoid chronic use, unless other alternatives are not effective and patient can take gastroprotective agent
Vasodilators	C01D, C04, C07F	68 (15.8)	Use with caution, may cause syncope
SSRIs	N06AB	53 (12.4)	Use with caution, may cause SIADH
Nonbenzodiazepine, benzodiazepine receptor agonist hypnotics	N05CF04, N05CF01, N05CF02, N05CF03	42 (9.8)	Avoid; adverse events in older adults such as delirium, falls, fractures, increased hospitalisations
Cardiovascular (amiodarone, digoxin, nifedipine with immediate-release, doxazosin)	C02CA04, C01AA05, C01AA02, C01AA52, C01AA08, C08CA05, C08GA01, C08CA55, C07FB03, C02CA01, C02LE01, G04CA03, C02AC01, N02CX02, S01EA04, C02LC01, C02LC51, C02AC0, C02AB, C02LB, C02AA0, C02LA01, C02LA51, C02LA71, C02AA52, C01BA03, C01BD07, C01BD01	21 (4.9)	Amiodarone: avoid as first-line therapy for AF unless patient has heart failure or left ventricular hypertrophy. Digoxin: avoid as first-line therapy for AF. Nifedipine: avoid, potential for hypotension and risk of precipitating myocardial ischaemia. Doxazosin: avoid as antihypertensive, risk of orthostatic hypotension
Association of chronic kidney disease and NSAIDs	M01A	21 (4.9)	Avoid; may increase risk of acute kidney injury and further decline of renal function

*AF* atrial fibrillation, *ATC* anatomical therapeutic chemical, *PIM* potentially inappropriate medication, *NSAIDs* non-steroidal anti-inflammatory drugs, *SIADH* syndrome of inappropriate antidiuretic hormone secretion, *SSRI* selective serotonin reuptake inhibitor

Details of all PIMs and comparison by sex/gender can be found in Table 1 in the Electronic Supplementary Material. Combined, the number of PIMs and proportions of patients with at least one PIM or with different PIM categories were comparable between men and women (at least one PIM: 76.2 vs. 77.5%; *p *= 0.760). Sex/gender differences were observed in terms of medication categories. The more frequent PIMs in women were antidepressants that should be avoided (4.5 vs. 0%; *p *= 0.010), SSRIs to be used with caution (15.6 vs. 6.9%; *p *= 0.008) and various psychotropic drugs to be avoided in patients with a history of fracture (4.5 vs. 0.6%; *p *= 0.025). PIMs more frequent in men were vasodilators to be used with caution (20.0 vs. 11.2%; *p *= 0.012).

## Discussion

Polypharmacy was very common in older patients followed regularly in family medicine in Switzerland, with three of five patients taking at least five drugs. Three-quarters of patients were prescribed at least one PIM according to Beers criteria. The most frequent PIMs were PPIs prescribed for a duration > 8 weeks, diuretics, benzodiazepines, aspirin for primary prevention of cardiac events and chronic use of NSAIDs. Prescription patterns markedly differed by sex/gender, with more PIMs found in women, who were prescribed more psychotropic drugs that should be avoided or used with caution with regards to their age and medical condition (fracture). Variations in prescription by physician were observed for cardiovascular drugs and vitamins.

### Polypharmacy and Prevalence of PIM

The prevalence of both polypharmacy and PIM was comparable to findings in recent data from Switzerland [4] but higher than previous estimates [3, 9, 10]. Participants in our study were older than in previous studies (> 75 years in our study vs. > 65 years). Polypharmacy tends to increase with age, and many Beers criteria start to apply systematically at the age of 75 years (e.g. chronic use of NSAIDs, dabigatran or prasugrel) or 80 years (aspirin for primary prevention). Our study highlights the high prevalence of benzodiazepines, non-benzodiazepine/benzodiazepine receptor agonist hypnotics, specific cardiovascular drugs, oral non-cyclooxygenase-selective NSAIDs for chronic use and SSRIs. Important differences exist between previous studies conducted in Swiss patients and our study. For example, aspirin for primary prevention of cardiac events by patients aged > 80 years and vasodilators were not reported in other claim-based studies, which do not have access to clinical information. By contrast, all these items were very common in our patient population, which may explain some of the differences in PIM prevalence [3, 10].

Among patients aged ≥ 75 years followed in family medicine, important differences in prescription patterns were observed by sex/gender. Men received more cardiovascular prevention drugs, whereas women received more mineral supplements and antidepressants, despite a similar prevalence of cardiovascular or psychological conditions in men and women. Cardiovascular drugs were also less prescribed in women. While some of these differences may still reflect true diagnosis prevalence differences, further attention should be given to potential under- or overdiagnosis of specific conditions in older patients, based on well-documented medical gender bias [15, 25].

In contrast with previous studies in which older women were prescribed more PIMs [8, 12, 13], we did not identify major differences in overall PIM prevalence. However, the sex/gender differences we found in the type of PIMs echo the differences found in prescription patterns: women were more likely to have PIMs related to antidepressants, and men were more likely to have PIMs related to vasodilators. Larger studies exploring the reasons for increased or different PIMs in women hypothesise on multiple biological and social factors. Sex differences in the prevalence of conditions may explain discrepancies in drug prescriptions (and therefore the risk of PIM) and may imply a different navigation of the health system and the number of health providers involved (increasing the risk of PIMs). Social factors include gender bias in diagnosis and treatment for similar conditions and the intersection of gender with other social factors, such as education, living conditions, communication modes and healthcare provider–patient interactions [14, 19].

### Limitations of the Study

Our analysis of potential inappropriateness was based solely on the Beers criteria. We included patients who consulted at least twice during the last year, which may have biased the sample towards patients who consult often and use more medication. Also, physicians participating in the cluster-randomised trial may not be fully representative of all Swiss physicians, although we tried to limit inclusion criteria as much as possible to align with the pragmatic nature of the trial. Some medical conditions (e.g. tobacco use, obesity) were only counted if listed in the medical file as a diagnosis, so may be underestimated. Creatinine clearance was not noted for patients with chronic kidney disease, which may have led to some NSAID use being misclassified as potentially inappropriate. Also, the distinction between primary and secondary prevention relied on the cardiovascular diagnoses mentioned in the medical file, which may also have been underreported. Overall, whereas we acknowledge the potential for misclassification for PIMs that require specific conditions, we believe the quality of the clinical information provided for these patients as part of an intervention trial to be better than that of routine health records or claims data. Finally, drug prescription is not equivalent to drug use, as patients may never start the prescribed drug or may stop it prematurely. This may have led to overestimation of polypharmacy but not the estimation of potentially inappropriate prescriptions considering that a drug not taken is still potentially inappropriately prescribed.

### Clinical Implications

This study highlighted the existing challenges in the medication of older patients in Switzerland in terms of PIM and polypharmacy. Polypharmacy based on the number of medications is not necessarily inappropriate, considering that patients with several diagnoses and comorbidities may require multiple medications that may all be clinically indicated. However, the risk of PIMs increases with the number of prescribed drugs. Tools to reduce PIMs, such as the PRISCUS list [26], Beers criteria [6], the Screening Tool of Older People’s Prescriptions (STOPP) and Screening Tool to Alert to Right Treatment (START) criteria [7], exist, but studies have shown that family physicians do not necessarily use them because of negative views [27]. Although physicians are aware of PIM and polypharmacy [27], medication they consider as potentially inappropriate does not necessarily match established criteria such as the PRISCUS list.

Interestingly, only a limited number of medication classes were involved in most PIMs. In this context, targeted information on the most prevalent PIM categories, for example PPIs, diuretics, benzodiazepines, and aspirin for primary prevention (Table 3), could be more efficient than lengthy deprescription lists. Including some deprescription advice in the ‘top five’ lists as promoted by the ‘Choosing wisely’ campaign is probably promising, but more effort is needed for these recommendations to be known to and applied by physicians [28]. Furthermore, prescription habits for specific drugs such as PPIs, NSAIDs, benzodiazepines and z-drugs must evolve to include limited treatment durations. We highlighted some differences in prescription habits by sex/gender, which suggested a need for physicians to reflect on their potential implicit gender biases in diagnosis and treatment. Indeed, targeted information on the most prevalent PIM categories, differentiating men and women, could be more efficient than lengthy deprescription lists. Such an approach should be further tested within deprescription trials.

## Conclusions

Both polypharmacy and potentially inappropriate prescribing were very common in older patients followed in family medicine in Switzerland. Interestingly, only a limited number of medication classes were involved in most PIMs, and patterns varied by sex/gender. In this context, simple deprescription lists targeting the most frequently inappropriately prescribed drugs according to patient sex/gender could prove more useful than lengthy generic advice to reduce potentially inappropriate prescriptions.

## Electronic supplementary material

Below is the link to the electronic supplementary material.

Supplementary material 1 (PDF 973 kb)
