# Supplementary material for: Drug Prescription in Older Swiss Men and Women Followed in Family Medicine
Source: Drugs Real World Outcomes. 2019 Dec 17;7(1):87–95. doi: 10.1007/s40801-019-00175-6 (PMC7060976; doi:10.1007/s40801-019-00175-6)
Supplement: Supplementary file 1 — Supplementary material 1 (PDF 973 kb) [file 40801_2019_175_MOESM1_ESM.pdf]

Supplementary material

Article title: Drug prescription in older Swiss men and women followed in family medicine
Journal name: Drugs – Real World Outcomes
Author names: David Schnegg, Nicolas Senn, Olivier Bugnon, Joëlle Schwarz, Yolanda Mueller
Corresponding author: Yolanda Mueller, Department of Family Medicine / Center for Primary Care and Public Health (Unisanté), University of Lausanne, Lausanne, Switzerland. Email: Yolanda.mueller@unisante.ch

Table 1. Potentially inappropriate medication in older adults included in AGE3 study, by sex/gender, based on American Geriatrics Society 2015 Beers Criteria Update

Category	Number of patients	% (N=429)	Women (N=269)	%	Men (N=160)	%	p	Drugs	ATC code or class	Number of prescriptions	% (N=429)
Medications that should be avoided in most older adults											
**Gastrointestinal**	100	23.1%	61	22.7 %	39	24.4%	0.687				
								Proton-pump inhibitors >8wks*	A02BC	99	23.1%
								Metoclopramide	A03FA01	6	1.4 %
**Benzodiazepines**	92	21.5%	61	22.7%	31	19.4%	0.420				
								Lorazepam	N05BA06N 05BA56	43	10.0%
								Oxazepam	N05BA04	25	5.8%
								Alprazolam	N05BA12	7	1.6%
								Clorazepate	N05BA05	6	1.4%
								Triazolam	N05CD05	5	1.2%
								Clonazepam	N03AE01	4	0.9%
								Flurazepam	N05CD01	3	0.7%
								Temazepam	N05CD07	1	0.2%

								Diazepam	N05BA01N 05BA17	1	0.2%
**Non-cyclooxygenase-selective NSAIDs, oral**	71	16.6	49	18.2%	22	13.8%	0.229				
								Ibuprofen	M01AE01	39	9.1%
								Diclofenac	M01AB05	14	3.3%
								Naproxen	M01AE02 M01AE52 M01AE56	10	2.3%
								Mefenamic acid	M01AG01	5	1.2%
								Etodolac	M01AB08	3	0.7%
								Piroxicam	M01AC01	1	0.2%
**Nonbenzodiazepine, benzodiazepine receptor agonist hypnotics**	42	9.8%	24	8.9%	18	11.3%	0.433				
								Zolpidem	N05CF02	37	8.6%
								Zopiclone	N05CF01	6	1.4%
**Cardiovascular**	21	4.9%	9	3.4%	12	7.5%	0.054				
								Amiodarone	C01BD01	15	3.5%
								Digoxin	C01AA05	4	0.9%
								Nifedipine, immediate release	C08CA05	3	0.7%
								Doxazosin		1	0.2%
**Anticholinergics**	9	2.1%	4	1.5%	5	3.1%	0.252				
								Scopolamine	A03BB01	5	1.2%
								Hydroxyzine	N05BB01	3	0.7%
								Diphenhydramine	R06AA02	1	0.2%
**Antithrombotics**	0	0.0%									

**Anti-infective**	2	0.5%	0	0.0%	2	0.7%	1.000	Long-term nitrofurantoin	J01XE01	2	0.5%
**Antidepressants**	11	2.6%	11	4.1%	0	0.0%	0.010	Paroxetine	N06AB05	7	1.6%
								Amitriptyline	N06AA09	2	0.5%
								Doxepin (>6mg/d)	N06AA12	1	0.2%
								Trimipramine	N06AA06	1	0.2%
**Barbiturates**	0	0.0%									
**Antipsychotics**								8 patients (1.9%), rationale impossible to assess			
**Endocrine**	1	0.2%	0	0.0%	1	0.4%	1.000	Estradiol	G03CA03	1	0.2%
**Subtotal**	240	55.9%	152	56.5%	88	55.0%	0.761				
**Condition** (ICPC-2 code)	Medications that should be avoided with specific disease or syndrome because they could worsen the condition.										
**Chronic kidney disease** (any creatinine clearance; specific variable "renal insufficiency"))	21	4.9%	16	6.0%	5	3.1%	0.199	NSAIDs	M01A	21	4.9%
**Heart failure** (k77)	19	4.4%	9	3.5%	10	6.3%	0.157	NSAIDs	M01A	17	4.0%
								Diltiazem	C08DB01	1	0.2%
								Verapamil	C08DA01	1	0.2%
**History of fractures** (a29, l72, l73, l74, l75, l76)	13	3.0%	12	4.5%	1	0.6%	0.025	Benzodiazepines	N05BA, N05CD	8	1.9%
								Nonbenzodiazepine, benzodiazepine receptor agonist hypnotics	N05CF	3	0.7%
								Anticonvulsants	N03	2	0.5%
								Tricyclic antidepressants	N06AA	2	0.5%

								Opioids	N02A	1	0.2%
								SSRIs	N06AB	1	0.2%
**Chronic seizures or epilepsy** (n88)	1	0.2%	1	0.4%	0	0.0%	1.000	Olanzapine	N05AH03	1	0.2%
**Delirium, insomnia** (p71)	0										
**Dementia or cognitive impairment** (p70)	1	0.2%	1	0.4%	0	0.0%	1.000	Antipsychotic	N05AH	1	0.2%
**Parkinson's disease** (n87)	1	0.2%	1	0.4%	0	0.0%	1.000	Antipsychotic	N05AH	1	0.2%
**Gastric or duodenal ulcer** (d85, d86)	0										
**Lower urinary tract symptoms, benign prostatic hyperplasia** (u05, u07, u08, u13, y85)	0										
**Subtotal**	52	12.1%	36	13.4%	16	10.0%	0.299				
	Medications that should be used with caution by older adults										
Diuretics	122	28.4%	72	26.8%	50	31.3%	0.319	Use with caution, may cause SIADH	C03		
Aspirin for primary prevention of cardiac events in 80+ years old	85	19.8%	49	18.2%	36	22.5%	0.282	Use with caution by patients aged ≥80 years	B01AC		
Vasodilatators	62	14.5%	30	11.2%	32	20.0%	0.012	Use with caution, may cause syncope	C01D, C04, C07F		
SSRIs	53	12.4%	42	15.6%	11	6.9%	0.008	Use with caution, may cause SIADH	N06AB		
Serotonin and norepinephrine	12	2.8%	8	3.0%	4	2.5%	0.773	Use with caution, may cause SIADH	N06AX16, N06AX21,		

reuptake inhibotrs (SNRI)									N06AX17, N06AX23		
Mirtazapine	11	2.6%	6	2.2%	5	3.1%	0.571	Use with caution, may cause SIADH	N06AX11		
Antipsychotics	10	2.3%	7	2.6%	3	1.9%	0.629	Use with caution, may cause SIADH	N05A		
Tricyclic antidepressants	5	1.2%	5	1.9%	0	0.0%	0.083	Use with caution, may cause SIADH	N06AA N06CA		
Dabigatran	1	0.2%	1	0.4%	0	0.0%	1.000	Use with caution if >75 years or chronic kidney disease	B01AE07		
Carbamazepine	1	0.2%	0	0.0%	1	0.4%	1.000	Use with caution, may cause SIADH	N03AF01		
**Subtotal**	**245**	**57.1%**	**154**	**57.3%**	**91**	**56.9%**	**0.940**				
Total	**329**	**76.7%**	**205**	**76.2%**	**124**	**77.5%**	**0.760**				
**Subtotal drug-drug interactions at risk by older patients**	**26**	**6.1%**	**13**	**4.8%**	**13**	**8.1%**	**0.209**				
**Subtotal using drugs while having a reduced creatinine clearance**	**23**	**5.4%**	**15**	**5.6%**	**8**	**5.0%**	**1.000**				

***** IPP : includes 17 patients on NSAIDs, 13 on cortocosteroids and 1 patient on both

One patient can receive more than one drug of single category (for example amiodarone and digoxine in “cardiovascular”)

Abbreviations: ATC: Anatomical Therapeutic Chemical (ATC); ICPC: International Classificaiton of Primary Care; NSAIDS: Non-steroidal anti-inflammatory drugs; SIADH: syndrome of inappropriate antidiuretic hormone secretion; SSRI: Selective serotonin reuptake inhibitors
